# Leveraging Mobile Phones for Monitoring Risks for Noncommunicable Diseases in the Future

**DOI:** 10.2196/jmir.7622

**Published:** 2017-05-05

**Authors:** Jennifer A Ellis

**Affiliations:** ^1^ Bloomberg Philanthropies New York, NY United States

**Keywords:** mHealth, low- and middle-income countries, noncommunicable diseases, health systems strengthening

One of the biggest contributors to preventable deaths isn't a health problem but a record-keeping problem—and it is one that can be solved. [1]

Noncommunicable diseases (NCDs) account for two-thirds of deaths globally. The World Health Organization estimates that 67% of 56 million deaths that took place globally in 2012 were due to NCDs, in particular cardiovascular diseases, cancers, chronic respiratory diseases, and diabetes [2]. Three-quarters of these deaths occurred in low- and middle-income countries (LMICs) and nearly half of deaths occurred in persons younger than 70 years [2]. The World Health Organization projects a rise in the number of NCD deaths from 36 million in 2008 to 55 million by 2030, of which 15.4 million are projected to be in people younger than 70 years, if effective steps are not taken to curb the pandemic [3,4].

Four key risk factors which have been stated to be responsible for a majority of NCDs are tobacco use, sodium intake, sedentary lifestyle, and excessive use of alcohol—all behavioral and largely modifiable [5]. These factors, as well as loss of employment due to NCD-related disability and the long duration and complexity of NCD treatment, pose additional challenges to poverty reduction and sustainable development [6].

In 2015, Bloomberg Philanthropies and the Australian Government launched an ambitious new effort to redress the serious need for improved data about why people die. The Data for Health Initiative [7] is a US $100 million program that aims to provide better health data, accelerate global progress, and provide technical resources for more than 1 billion people in more than 20 LMICs in Africa, Asia, and Latin America. Reliable data are essential to make effective health policies, measure the success of public health interventions, and prioritize research. To reach the Initiative’s goal, Bloomberg Philanthropies has brought together a group of leading partners that includes the Johns Hopkins University Bloomberg School of Public Health, the CDC Foundation, Vital Strategies, the University of Melbourne, and the World Health Organization.

The day-to-day things that are killing people in large numbers are largely non communicable diseases and injuries... Yet governments, donors, and global health leadership are not responding... [8].

Serious gaps exist between health care spending and need—particularly in LMICs. In order to understand where investment has the most potential, we need detailed and dependable information about what is killing people. Illnesses like cancer and diabetes are becoming less fatal in higher income countries thanks to investment in prevention and treatment. Without the same in LMICs, chronic diseases are projected to cost US $21.3 trillion over the next two decades—and countless lives [8].

Many LMICs seek cost-effective methods to obtain timely and quality NCD risk factor data that can be used to inform resource allocation, policy development, and assist in the evaluation of NCD trends over time. Mobile phone technology is omnipresent in many LMICs and presents an untapped opportunity for using population-level health surveys. The Data for Health Initiative is working to assess, harness, and roll out the use of mobile phone technology in LMICs as a cost-effective method for the rapid collection of quality NCD risk factor data. Countries engaged in the Initiative expressed interest in improving their public health data and present particularly great potential for gains. There are, however, critical gaps in understanding the ways by which mobile phone surveys could be a cutting-edge aid in the collection of NCD data in LMICs.

This special Theme Issue of JMIR addresses these critical gaps through an examination of the relevant extant literature, analysis of the myriad ethical issues surrounding the use of mobile phones to collect personal health data in LMICs, methodological challenges, conceptual challenges of turning data into international- and national-level policies, and the efforts needed for using mobile phone technology to create a research agenda for NCDs, public health interventions, and strengthening health systems in LMIC contexts.

Current methods of data collection in LMICs rely on the use of household surveys to monitor disease burden in countries, prioritize resource allocation, and evaluate public health policies. In LMICs, household surveys typically rely on face-to-face interviews conducted at the respondent’s household. Maintaining up-to-date data based on household surveys is difficult, however, since household surveys bear high costs, particularly in personnel and transportation [9], which means such surveys cannot be conducted too frequently. Additionally, household surveys require considerable amounts of time for data collection, data management, and data analysis which impedes the speed at which data become publically available. Mobile phone technology presents a potentially viable option for more frequent surveillance of population health, one that will permit more timely evaluation of implemented public health policies and response to public health emergencies.

To address the high costs and time requirements associated with household surveys, higher-income countries have developed and employed telephone and mobile phone surveys to collect population-level estimates of health [9]. The global spread of mobile phone ownership and access provides new opportunities to leverage mobile health technologies and communication channels to revolutionize current methods of data collection in LMICs. Mobile phone surveys involve interviewing respondents over their own personal mobile phone through the use of short message service, interactive voice response, and computer-assisted telephone interviews. This JMIR Theme Issue explores and compares the challenges and benefits of each of these survey modalities as well as the current landscape of mobile phone survey technology being used for population-level data collection in LMICs.

## Abbreviations

NCD: noncommunicable disease
LMIC: low- and middle-income country
